# Adequate sample size for developing prediction models is not simply related to events per variable

**DOI:** 10.1016/j.jclinepi.2016.02.031

**Published:** 2016-08

**Authors:** Emmanuel O. Ogundimu, Douglas G. Altman, Gary S. Collins

**Affiliations:** Centre for Statistics in Medicine, Nuffield Department of Orthopaedics, Rheumatology & Musculoskeletal Diseases, Botnar Research Centre, University of Oxford, Windmill Road, Oxford OX3 7LD, UK

**Keywords:** Events per variable, Cox model, External validation, Predictive modeling, Sample size, Resampling study

## Abstract

**Objectives:**

The choice of an adequate sample size for a Cox regression analysis is generally based on the rule of thumb derived from simulation studies of a minimum of 10 events per variable (EPV). One simulation study suggested scenarios in which the 10 EPV rule can be relaxed. The effect of a range of binary predictors with varying prevalence, reflecting clinical practice, has not yet been fully investigated.

**Study Design and Setting:**

We conducted an extended resampling study using a large general-practice data set, comprising over 2 million anonymized patient records, to examine the EPV requirements for prediction models with low-prevalence binary predictors developed using Cox regression. The performance of the models was then evaluated using an independent external validation data set. We investigated both fully specified models and models derived using variable selection.

**Results:**

Our results indicated that an EPV rule of thumb should be data driven and that EPV ≥ 20 ​ generally eliminates bias in regression coefficients when many low-prevalence predictors are included in a Cox model.

**Conclusion:**

Higher EPV is needed when low-prevalence predictors are present in a model to eliminate bias in regression coefficients and improve predictive accuracy.

**What is new?**

**Key findings**
• The use of a rule of thumb for selecting events per variable (EPV) should be study dependent.
• Convergence in Cox models depends more on the severity of low prevalence in binary predictors and much less on low EPV.
• Higher EPV is needed when low-prevalence predictors are present in a model to eliminate bias in regression coefficients and improve predictive accuracy.

**What is the implication and what should change now?**
• EPV ≥ 20 should be considered when a data set includes low-prevalence binary predictors - if EPV ≥ 20 cannot be guaranteed, then the use of the penalized likelihood approach should be considered.

## 1 Introduction

When multivariable prediction models are developed, the sample size is often based on the ratio of the number of individuals with the outcome event to the number of candidate predictors (more precisely, the number of parameters), referred to as the events per variable (EPV). Models developed from data sets with too few outcome events relative to the number of candidate predictors are likely to yield biased estimates of regression coefficients. They lead to unstable prediction models that are overfit to the development sample and perform poorly on new data. Simulation studies of prediction models developed using both logistic regression and Cox regression have suggested minimum EPV values of between 5 and 20 for reliable results [1], [2], [3], [4], [5]. An EPV of 10 is widely advocated as the rule of thumb for multivariable logistic and Cox regression analyses.

Through their influential work, Peduzzi et al. [1], [3], [4] encouraged the use of the 10 EPV rules for both logistic- and Cox regression–based prediction models. However, there were limitations to the design of their simulation studies, particularly with respect to prediction. They emphasized accuracy and precision of the regression coefficients, rather than the measures of predictive ability. The studies were also based on a relatively small data set of 673 individuals (252 of whom had the outcome event) and only considered one prediction model that contained seven predictors (six binary and one ordinal). Predictors were not selected, either before or during the model building. Although these highly cited simulation studies have raised awareness of the importance of the number of outcome events relative to the number of predictors, the limited scenarios examined cast doubt on the generalizability of their findings.

Subsequent simulation studies have examined more complex scenarios by altering the number of predictors in fixed regression models. Some have suggested that the 10 EPV rules can be relaxed [5], and others that no single EPV rule of thumb can guarantee accurate estimates of regression coefficients [6]. However, these studies have also focused on establishing a recommended minimum EPV in the context of stable regression coefficients, without considering the predictive ability of the model. They have also not considered the generalizability of the findings to real-life settings, for example, when investigators are confronted with many candidate predictors and must choose a subset to include in their final prediction model [7].

Studies examining the influence of backward elimination for predictor selection have shown that the regression coefficients from a logistic regression model may have considerable bias, particularly in small samples [8]. Studies examining the effect of EPV on the development of regression models have therefore tended to use small single data sets and have focused on accurate parameter estimation of regression coefficients. They have offered limited insights into the effect on the predictive performance of the model (e.g., calibration and discrimination).

The presence of low-prevalence binary predictors can induce the problem of complete (or quasi) separation in logistic regression [9], [10] or monotone likelihood in Cox regression [11]. These problems may be noticed in an individual study when parameters and standard errors are too large to be useful. The parameter estimates are not unique and depend on trivial issues like the settings of software used for the analysis. While keeping other design factors constant, the probability of separation or monotone likelihood is lower with higher EPV values.

Heinze and Schempe [11] extended the modified likelihood method of Firth [12] to circumvent monotone likelihood problems in the estimation of parameters from Cox model with low-prevalence predictors. However, applied researchers still typically do not apply Firth's correction when fitting a Cox regression model. We focused on this practice and investigated the EPV requirement for parameter estimates and predictive accuracy in the presence of low-prevalence but highly prognostic binary predictors.

We conducted a resampling study using a large general practice data set, comprising over 2 million anonymized patient records, to examine the relationship between EPV, accuracy of regression coefficients, and predictive ability using Cox regression. We investigated scenarios with both fully prespecified models and models derived from the data using automated variable selection. We examined the stability and precision of the regression coefficients and their effect on the models' predictive performance (e.g., the *c*-index, *D*-statistic, and *R*^2^). We also examined the effect of EPV in the development of a prediction model on the model's subsequent performance using a separate large external validation data set.

## 2 Data and methods

### 2.1 Study data: The Health Improvement Network

The Health Improvement Network (THIN) is a large database of anonymized electronic health care records collected from general-practice clinics around the United Kingdom (England, Scotland, Wales, and Northern Ireland). The THIN database currently contains medical records from approximately 4% of the United Kingdom population. We used clinical information from 2,084,445 individuals, aged 30 to 84 years, registered between June 1994 and June 2008 from 365 general practices. The characteristics of the THIN data set are summarized in Table 1. Twelve variables were considered: one categorical [smoking status (SMK); four categories], four continuous [age, systolic blood pressure (SBP), body mass index (BMI), and ratio of total serum cholesterol to high-density lipoprotein (RATIO)], and seven binary [sex, diagnosis of type diabetes (TYPE2), rheumatoid arthritis (BRA), atrial fibrillation (BAF), renal disease (RENAL), treated hypertension (HYPER), and family history of coronary heart disease (FHCVD)]. Because of the low prevalence of some of the SMK categories, we combined nonsmokers and former smokers as “nonsmokers” and the rest as “smokers.” The primary outcome was cardiovascular disease (CVD), which was experienced by 93,564 individuals in the THIN data set.

Prediction models were developed using the entire THIN data set, omitting individuals from Scotland (THIN_*d*_). The individuals from Scotland (THIN_*v*_) were used to validate the prediction models in an external validation setting. The sample sizes of the development and validation data sets were 1,973,511 individuals (88,312 CVD events) and 110,934 individuals (5,252 CVD events), respectively.

### 2.2 Resampling scheme and models

#### 2.2.1 Resampling scheme

One thousand random samples with replacement were drawn from the THIN development data set (THIN_*d*_). Events rate in each of the random samples are fixed by randomly sampling separately from those who did and did not experience the event of interest. The event rate in the entire development data set is 4.5%. Specifically, the number of individuals with the event and without the event was exactly the same in each of the random sample for a given EPV setting. The EPV values considered are 2, 5, 10, 15, 20, 25, and 50.

#### 2.2.2 Models

Fixed prespecified models containing 3, 5, 7, 10, and 12 predictors were examined. This approach allowed the relationship between the predictors and the outcome variable to be maintained across independent simulations. As the models were fully nested, they could easily be compared. The use of fractional polynomials suggested that the continuous predictors could be modeled linearly. The three-predictor model contained BMI, age, and sex. The five-predictor model contained BMI, age, sex, SBP, and RATIO. These variables were chosen based on their importance in the model fitted to the THIN_*d*_ data set. We defined variable importance using the standardized regression coefficients of the fitted model. The standardized coefficients were ordered by absolute value, and this order was used to select the variables for the models (see Table 2). Thus, the three-predictor model contains variables with the strongest effects (BMI, age, and sex). Binary predictors were added to the models using the same approach, forming the 7-predictor model (5-predictor model with HYPER and TYPE2), 10-predictor model (7-predictor model with SMK, FHCVD, and BRA), and 12-predictor model (10-predictor model with BAF and RENAL).

In addition to examining the effect of the EPV on the prespecified models, we also examined models in which the predictors were selected using the backward elimination variable selection method. A pilot simulation to assess the performance of various models showed that all the models converged (nonconvergence of models is indicated by error message in the partial likelihood maximization algorithm) for the five-predictor model. Therefore, we considered this model along with 10 randomly generated noise variables from a normal distribution with a mean of 0 and a variance of 1. The choice of 10 noise variables was arbitrary. Using backward elimination, variables were omitted until all the variables retained in the model were statistically significant at a significance level of either 0.05 or 0.157 [the Akaike information criterion (AIC)]. The performance of these models was also evaluated using the external validation Scottish data set (THIN_*v*_). Instances in which convergence was not achieved were excluded and not replaced in the analysis. The frequency of nonconvergence was recorded for each setting.

Analysis of the full THIN development data set (*n* = 1,973,511) for models containing 3, 5, 7, 10, and 12 predictors provided the “true” regression coefficients and performance measures. The models developed from smaller samples and with fewer variables were evaluated against these “true” values. Given the size of the development data set (THIN_*d*_), all the variables were statistically significant in the models. The EPV values for the models with 3, 5, 7, 10, and 12 predictors generated from the THIN_*d*_ data were 29,437, 17,662, 12,616, 8,831, and 7,359.

The analysis was carried out using the statistical software R (version 3.0.3).

### 2.3 Analysis of simulations

#### 2.3.1 Regression coefficients

We examine the performance of regression coefficients using the guidance by Burton et al. [13]. The accuracy of each regression coefficient (${\hat{\beta }}_{k}$) was assessed by calculating the signed percent relative bias, $100\times \left({\bar{\hat{\beta }}}_{k}-\beta \right)/\beta$, where ${\bar{\hat{\beta }}}_{k}=\sum_{k=1}^{m}{\hat{\beta }}_{k}/m$, *m* is the number of models that converged and *β* is the “true” regression coefficients from the models. The precision of the regression coefficients was assessed by calculating the ratio of the model and empirical simulation variance, which is an indicator of the large sample properties of a prediction model. The model variance was calculated by $\sum s^{2}/m$, where *s* is the model-based standard error. The empirical simulation variance was calculated by $\sum {\left({\bar{\hat{\beta }}}_{k}-\beta \right)}^{2}/\left(m-1\right)$. If this ratio is substantially different from 1, the Cox model concerned is unlikely to have large sample properties [1]. We also calculated the proportion of simulations in which all the variables were statistically significant (*P* < 0.05) and the coverage of the 95% confidence interval (i.e., the proportion of times the confidence interval contained the “true” performance value).

#### 2.3.2 Model predictive performance

Predictive performance was evaluated on both the data used to develop the prediction models (THIN_*d*_), referred to as apparent performance, and a separate validation data set (THIN_*v*_), referred to as external validation. For each value of the EPV, we calculated the concordance index (discrimination), prognostic separation measured by the D-statistic [14] and measures of explained variation (*R*^2^) *R*_*rs*_ (derived from the D-statistic), and *R*_*oxs*_ [15] at each iteration in the simulation.

We also investigated the behavior of the calibration slope using the external validation sample. The calibration slope was estimated as the regression coefficient in a Cox model with one term, the prognostic index [16]. For each performance measure, we calculated the percent relative bias and the root mean square error (RMSE), defined as $\sqrt{\frac{1}{m}\sum_{k=1}^{m}{\left({\hat{\beta }}_{k}-\beta \right)}^{2}}$.

## 3 Results

The results of the Monte Carlo simulations for each model are reported in Table 3 and Tables A.1.1–A.1.5 at www.jclinepi.com in the online supplementary material.

Low-prevalence binary predictors were added to the models sequentially in order of importance to form the 7-, 10-, and 12-predictor models. The frequency of model nonconvergence increased with the addition of these variables. As expected, increasing the EPV alleviated nonconvergence and resulted in more stable parameter estimates. Only three models failed to converge under EPV = 2 in the three-predictor model (Table 3). One variable also failed at EPV = 2 in the five-predictor model. In contrast, 419 models failed to converge in the seven-predictor model at EPV = 2, which was made up of the five-predictor model with two binary predictors. The frequency of failures decreased as EPV increased. Only seven models converged in the 12-predictor model at EPV = 2 (see Tables A.1.1–A.1.4 at www.jclinepi.com in the Appendix).

### 3.1 Bias and significance testing

**Fig. 1:**
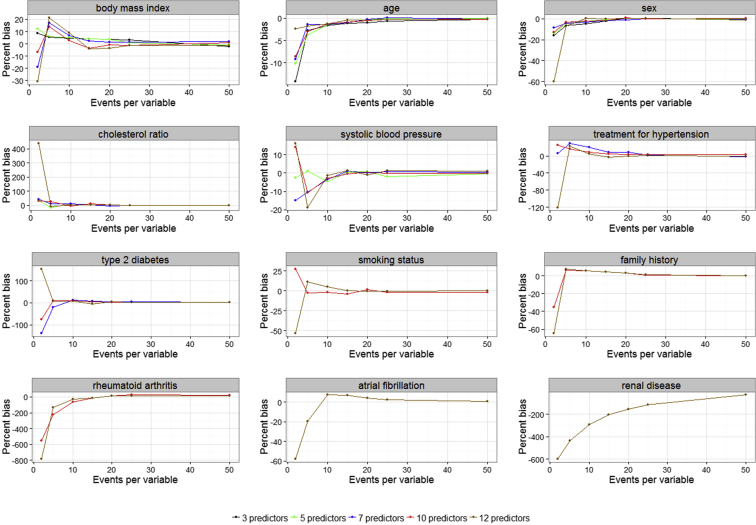
Number of events per variable and average percent relative bias for the variables in the data set.

The regression coefficients can be summarized based on the models they were derived from. For example, we can compare the percentage bias for BMI obtained from the 3-, 5-, 7-, 10-, and 12-predictor models. As TYPE2 was only included in the 10- and 12-predictor models, there are only two comparisons for this variable. The bias in BMI was highest when it was included in the 12-predictor model. Using the recommended EPV = 10 produced negligible bias in the 3- and 5-predictor models. However, EPV = 20 was required to achieve the same level of bias in the 7-, 10-, and 12-predictor models. Age and sex showed similar performances (see Fig. 1). Fig. 1 shows that the 12-predictor model performed poorly, in term of bias, for all the variables at low EPV.

**Fig. 2:**
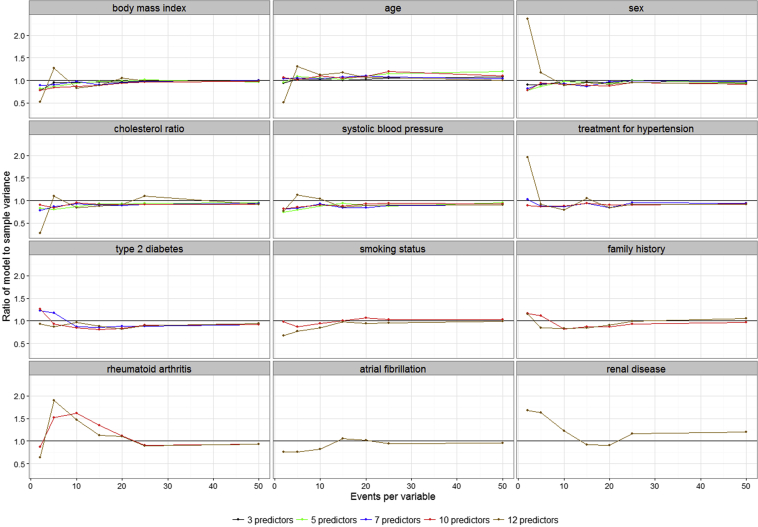
Ratio of model variance to sample variance for the variables in the data set.

Fig. 2 shows the ratio of model to sample variance for the variables in the fitted models. At EPV = 10, BMI, age, and sex had better precision in the 3- and 5-predictor models than in the 7-, 10-, and 12-predictor models. The ratio approached 1 when EPV > 10 in the 7-, 10-, and 12-predictor models. The precision of the two extremely low-prevalence binary predictors, BAF and RENAL, was poor even at EPV > 20. For HYPER, TYPE2, SMK, FHCVD, and BRA, EPV ≥ 20 was required to achieve the valid large sample properties of the fitted Cox model. This property could not be achieved for all the covariates in the model at EPV = 2.

**Fig. 3:**
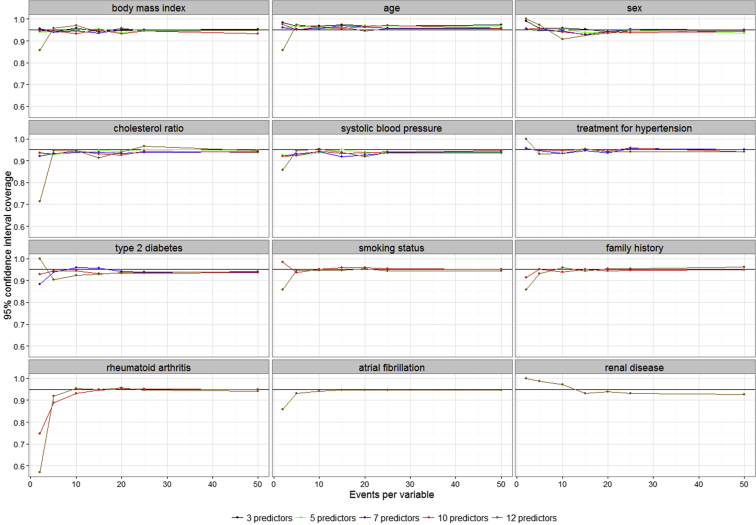
Proportion of simulations in which the 95% confidence interval about the simulated regression coefficient includes the “true” value for the variables in the data set.

Fig. 3 shows the 95% coverage probabilities for the variables in the models. The coverage was poor for all the variables in the 10- and 12-predictor models at EPV = 2. This was expected, as only 7 of the 1,000 models converged. We however caution our readers on setting up a rule of thumb for EPV in the case of severe model nonconvergence such as this. This is because counterintuitive results can be obtained if the selected results are not a random sample from all the models under consideration for the scenario. EPV = 10 was adequate for obtaining 95% coverage for other scenarios. Table 3 and Tables A.1.1–A.1.4 at www.jclinepi.com show how often the predictors were found to be significant at the 0.05 level of significance under the null hypothesis of no covariate effect. We based this calculation on the number of models that converged, not on the 1,000 replicates. The predictors were significant more often as EPV increased. The sequential addition of binary predictors did not have a specific effect on the significance of the predictors in the models before their addition.

### 3.2 Predictive performance

The performances of the two *R*^2^-type measures of predictive accuracy are as expected. *R*_*oxs*_, for example, is a measure of explained randomness and is given as $1-\exp\left(-2\left(l_{\beta }-l_{0}\right)/k\right)$, where *l*_*β*_ and *l*_0_ are the log partial likelihoods for the model with the covariates and the null model, respectively, and *k* is the number of events. For the same set of variables in a model, an increase in the number of events will lead to increase in this measure. On the other hand, for a fixed number of events, the difference between *l*_*β*_ and *l*_0_ also contributes to model performance. Fig. B.1.1 (Appendix) at www.jclinepi.com shows that *R*_*oxs*_ and *R*_*rs*_ improved as the number of events increases for the 3-, 5-, 7-, 10-, and 12-predictor models. When low-prevalence binary predictors are included in a model, the probability of model convergence is lowered. The parameters that converge may not converge to the maximum likelihood estimates, and the fit of the model is affected. As seen from Fig. B.1.1 at www.jclinepi.com, the 12-predictor model had the highest bias on the *R*^2^-type measures. In this case, we have extremely low-prevalence predictors, which affected the model fit. The difference between *l*_*β*_ and *l*_0_ is smaller than would be expected from a model that fits the data well for the same number of events, and the predictive accuracy of the model decreases.

Similar observation can be made about *c*-index and *D*-statistic, which are both measures of model discriminating ability. Fig. B.1.1 at www.jclinepi.com shows that EPV = 20 resulted in a better performance than EPV = 10. The corresponding RMSE for these measures, in contrast, showed that the three-predictor model had the largest errors. However, the scale of the error difference was small (in the range of 0.01). Again, the RMSE improved when EPV = 20.

### 3.3 External validation

The developed models did not perform as well on the Scottish data set in the external validation. As with the development data, the three-predictor model consistently outperformed the other models in terms of bias. However, the RMSE values were comparable. EPV = 10 was clearly a poor choice for these cases, and EPV ≥ 25 was a better choice (Fig. B.1.2 at www.jclinepi.com in the Appendix).

Table A.1.5 (Appendix) at www.jclinepi.com shows the frequency of variable selection for different EPV values when no low-prevalence binary predictors were considered. Backward elimination using the AIC selected the main predictors more often than backward elimination based on a significance level of 0.05. The strength of the selection methods improved as EPV increased. The probability of selecting all the main predictors simultaneously (the variable “all” in Table A.1.5 at www.jclinepi.com) was low at low EPV, but improved as EPV increased. The backward elimination method with AIC was superior to the *P*-value = 0.05 criterion in the selection of the five main predictors in the data set.

Fig. B.1.3 (Appendix) at www.jclinepi.com shows the comparison of the models generated by the two backward elimination methods to the true five-predictor model using the external validation data. The models generated using *P*-value = 0.05 as the inclusion criterion had slightly better predictive accuracy measures, based on bias and RMSE, than the models generated with the AIC. The true five-predictor model had the worst predictive accuracy. This observation is not surprising as the models developed through variable selection might have been overfitted. All the models were equivalent at EPV ≥ 25.

## 4 Discussion

Peduzzi et al. [3] recommended EPV = 10 for Cox models and noted that low prevalence of binary predictors aggravated EPV problems, such as model nonconvergence. We investigated the effect of EPV requirements on a range of binary predictors with varying prevalence using a large general-practice data set. We examined full (prespecified) models and models developed from backward selection methods using two variable inclusion criteria. The precision, significance, and predictive accuracy of the prespecified models were examined. Only the predictive accuracy of the models developed by variable selection was examined. The models were also evaluated using an external data set.

In general, the addition of low-prevalence binary predictors into an otherwise stable model required a higher EPV than the original model. For example, the bias in BMI was eliminated at 10 ≤ EPV ≤ 15 using the 3- and 5-predictor models. However, this bias was only removed from the 10- and 12-predictor models at EPV ≥ 20. As noted by Heinze and Schempe [11], the probability for the occurrence of monotone likelihood is likely to depend on sample size, censoring of survival times, magnitude of the relative risk associated with binary predictors, and the degree of balance in their distribution. The probability of monotone likelihood increases with increase in highly prognostic low-prevalence binary predictors but decreases as censoring probability decreases as we have shown here.

The EPV requirement for improved prediction depended on the measures used. The *R*_*oxs*_ required EPV ≥ 20 to achieve negligible bias, whereas the *R*_*rs*_ and D-statistic required EPV > 40 to achieve equivalent results. The three-predictor model consistently had less bias and a greater RMSE than the other models. However, the performance of these models on the external validation data was not significantly different in terms of bias or RMSE.

Overall, it is difficult to determine a definitive EPV value suitable for every situation. As a guide, EPV ≥ 20 should be considered when a data set includes low-prevalence binary predictors. If this cannot be guaranteed, then the use of the penalized likelihood approach of [12] is recommended, as it has been shown to reduce bias in parameter estimates on data with rare events [17].

Our study has some limitations. Although we used a large data set that reflected typical scenarios faced by researchers developing prediction tools, it did not permit a closer systematic evaluation of the roles of other features that real data may possess. For example, high regression coefficients and high correlations between predictors can affect the predictive accuracy of a Cox model. We included a number of low-prevalence binary predictors in our models as they are perceived to be important. Unsurprisingly, the problem of monotone likelihood and therefore a lack of model convergence abounded at low EPV values.

Another concern was that the simulation results were only based on models that converged, instances of nonconvergence were omitted. The modified likelihood method of Firth [12] has been suggested for overcoming the problem of monotone likelihood due to low EPV [17]. This estimator can be substituted for models that fail to converge in simulation settings that involve Cox and logistic models [18]. If prediction is the goal of data analysis, ridge regression may also used at low EPV values [16]. We have shown that EPV ≥ 20 ​ is required when low-prevalence predictors are present. The effect of modified likelihood and ridge regression methods in low-prevalence settings deserves further investigation.

## Figures and Tables

**Table 1:** Descriptive statistics for the predictors (*n* = 1,973,511)

Variable	Mean ± std. dev.	Frequency
Body mass index (BMI)	26.25 ± 4.41	
Age	48.66 ± 14.09	
Sex		Male: 0.49; female: 0.51
Cholesterol ratio (RATIO)	4.04 ± 1.31	
Systolic blood pressure (SBP)	131.84 ± 20.34	
Treatment of hypertension (HYPER)		No: 0.95; yes: 0.05
Type 2 diabetes (TYPE2)		No: 0.98; yes: 0.02
Smoking status (SMK)		Nonsmoker: 0.55; former smoker: 0.18
		Light smoker: 0.07
		Moderate smoker: 0.11
		Heavy smoker: 0.10
Family history of coronary		
Heart disease (FHCVD)		No: 0.96; yes: 0.04
Rheumatoid arthritis (BRA)		No: 0.99; yes: 0.01
Atrial fibrillation (BAF)		No: 0.99; yes: 0.01
Renal disease (RENAL)		No: 1.00; yes: 0.00

**Table 2:** Cox model with 12 covariates fitted to the THIN data

Predictor	Estimate $\left(\hat{\beta }\right)$	Standard error (SE)	$\text{Z}=\hat{\beta }/\text{SE}$
BMI	0.0233	0.0001	298.85
Age	0.0725	0.0003	258.37
Sex	0.4667	0.0068	68.86
RATIO	0.0410	0.0010	40.89
SBP	0.0069	0.0002	41.48
HYPER	0.2278	0.0071	32.04
TYPE2	0.5174	0.0137	37.75
SMK	0.3964	0.0181	21.92
FHCVD	0.8959	0.0391	22.90
BRA	0.2991	0.0265	11.27
BAF	0.5293	0.0490	10.80
RENAL	0.4919	0.0599	8.21

*Abbreviations:* THIN, The Health Improvement Network; BMI, body mass index; RATIO, cholesterol ratio; SBP, systolic blood pressure; HYPER, hypertension; TYPE2, type 2 diabetes; SMK, smoking status; FHCVD, family history of coronary heart disease; BRA, rheumatoid arthritis; BAF, atrial fibrillation; RENAL, renal disease.

**Table 3:** Number and percentage of occasions in which each variable was statistically significant at 0.05 level of significance using the three-predictor model

Variable	EPV = 2	EPV = 5	EPV = 10	EPV = 15	EPV = 20	EPV = 25	EPV = 50
	*N* (%)	*N* (%)	*N* (%)	*N* (%)	*N* (%)	*N* (%)	*N* (%)
Converged^a^	970	1,000	1,000	1,000	1,000	1,000	1,000
BMI	79 (8.1)	120 (12.0)	165 (16.5)	202 (20.2)	230 (23.0)	284 (28.4)	462 (46.2)
Age	732 (75.5)	991 (99.1)	1,000 (100.0)	1,000 (100.0)	1,000 (100.0)	1,000 (100.0)	1,000 (100.0)
Sex	36 (3.7)	138 (13.8)	267 (26.7)	399 (39.9)	478 (47.8)	574 (57.4)	852 (85.2)

*Abbreviations:* EPV, events per variable; BMI, body mass index.

a Number of models that converged out of 1,000 samples.
