# Supplementary material for: Adequate sample size for developing prediction models is not simply related to events per variable
Source: J Clin Epidemiol. 2016 Aug;76:175–82. doi: 10.1016/j.jclinepi.2016.02.031 (PMC5045274; doi:10.1016/j.jclinepi.2016.02.031)
Supplement: Appendix [file mmc1.pdf]

Supplementary material to: Adequate sample size for developing prediction models is not simply related to events per variable

Emmanuel O. Ogundimu* Douglas G. Altman Gary S. Collins

Centre for Statistics in Medicine, University of Oxford.

Appendix

A.1. **Tables A.1.1 - A.1.4: Number of occasion each variable was significant as the number of model predictors increases**

Table A.1.1: Number and percentage of occasions in which each variable was statistically significant at 0.05 level of significance using the five predictors model.

	EPV=2	EPV=5	EPV=10	EPV=15	EPV=20	EPV=25	EPV=50
Variable	*N*(%)	*N*(%)	*N*(%)	*N*(%)	*N*(%)	*N*(%)	*N*(%)
Converged[a]	999	1,000	1,000	1,000	1,000	1,000	1,000
BMI	84 (8.4)	88 (8.8)	110 (11)	174 (17.4)	184 (18.4)	208 (20.8)	380 (38.0)
AGE	807 (80.8)	997 (99.7)	1000 (100.0)	1000 (100.0)	1000 (100.0)	1000 (100.0)	1000 (100.0)
SEX	87 (8.7)	216 (21.6)	369 (36.9)	519 (51.9)	608 (60.8)	717 (71.7)	943 (94.3)
RATIO	72 (7.2)	86 (8.6)	105 (10.5)	107 (10.7)	141 (14.1)	141 (14.1)	196(19.6)
SBP	91 (9.1)	115 (11.5)	181 (18.1)	250 (25)	326 (32.6)	368 (36.8)	620 (62.0)

[a]Number of models that converged out of 1,000 samples.

*Correspondence to: Dr. E.O. Ogundimu, CSM, The University of Oxford, OXFORD, OX3 7LD, UK. E-mail address: emmanuel.ogundimu@csm.ox.ac.uk

Table A.1.2: Number and percentage of occasions in which each variable was statistically significant at 0.05 level of significance using the seven predictors model.

	EPV=2	EPV=5	EPV=10	EPV=15	EPV=20	EPV=25	EPV=50
Variable	N(%)	N(%)	N(%)	N(%)	N(%)	N(%)	N(%)
Converged [a]	581	884	989	998	999	999	1,000
BMI	54 (9.3)	91 (10.3)	121 (12.2)	170 (17.0)	181 (18.1)	213 (21.3)	343 (34.3)
AGE	508 (87.4)	884 (100.0)	989 (100.0)	998 (100.0)	999 (100.0)	999 (100.0)	1000 (100.0)
SEX	90 (15.5)	233 (26.4)	485 (49.0)	644 (64.5)	763 (76.4)	851 (85.2)	991 (99.1)
RATIO	57 (9.8)	85 (9.6)	108 (10.9)	136 (13.6)	140 (14.0)	182 (18.2)	267 (26.7)
SBP	55 (9.5)	141 (16.0)	224 (22.7)	301 (30.2)	362 (36.2)	443 (44.3)	710 (71.0)
HYPER	40 (6.9)	73 (8.3)	117 (11.8)	157 (15.7)	181 (18.1)	226 (22.6)	353 (35.3)
TYPE2	123 (21.2)	158 (17.9)	226 (22.9)	298 (29.9)	339 (33.9)	367 (36.7)	602 (60.2)

[a]Number of models that converged out of 1,000 samples.

Table A.1.3: Number and percentage of occasions in which each variable was statistically significant at 0.05 level of significance using the ten predictors model.

	EPV=2	EPV=5	EPV=10	EPV=15	EPV=20	EPV=25	EPV=50
Variable	N(%)	N(%)	N(%)	N(%)	N(%)	N(%)	N(%)
Converged [a]	146	532	794	895	959	986	999
BMI	19 (13.0)	44 (8.3)	135 (17.0)	210 (23.5)	280 (29.2)	350 (35.5)	587 (58.8)
AGE	144 (98.6)	532 (100.0)	794 (100.0)	895 (100.0)	959 (100.0)	986 (100.0)	999 (100.0)
SEX	30 (20.5)	199 (37.4)	492 (62.0)	707 (79.0)	869 (90.6)	924 (93.7)	998 (99.9)
RATIO	15 (10.3)	47 (8.8)	98 (12.3)	78 (8.7)	120 (12.5)	137 (13.9)	231 (23.1)
SBP	23 (15.8)	75 (14.1)	220 (27.7)	340 (38.0)	421 (43.9)	534 (54.2)	813 (81.4)
HYPER	10 (6.8)	69 (13.0)	135 (17.0)	188 (21.0)	225 (23.5)	296 (30.0)	482 (48.2)
TYPE2	35 (24.0)	101 (19.0)	218 (27.5)	341 (38.1)	428 (44.6)	497 (50.4)	734 (73.5)
SMK	20 (13.7)	134 (25.2)	319 (40.2)	475 (53.1)	604 (63.0)	736 (74.6)	957 (95.8)
FHCVD	45 (30.8)	192 (36.1)	465 (58.6)	630 (70.4)	770 (80.3)	868 (88.0)	987 (98.8)
BRA	53 (36.3)	97 (18.2)	90 (11.3)	109 (12.2)	107 (11.2)	139 (14.1)	165 (16.5)

[a]Number of models that converged out of 1,000 samples.

Table A.1.4: Number and percentage of occasions in which each variable was statistically significant at 0.05 level of significance using the 12 predictors model.

	EPV=2	EPV=5	EPV=10	EPV=15	EPV=20	EPV=25	EPV=50
Variable	N(%)	N(%)	N(%)	N(%)	N(%)	N(%)	N(%)
Converged [a]	7	73	264	389	483	608	883
BMI	1 (14.3)	8 (11.0)	53 (20.1)	97 (24.9)	168 (34.8)	246 (40.5)	528 (63.4)
AGE	6 (85.7)	73 (100.0)	264 (100.0)	389 (100.0)	483 (100.0)	608 (100.0)	833 (100.0)
SEX	2 (28.6)	22 (30.1)	182 (68.9)	338 (86.9)	445 (92.1)	591 (97.2)	833 (100.0)
RATIO	0 (0.0)	6 (8.2)	30 (11.4)	37 (9.5)	81 (16.8)	113 (18.6)	209 (25.1)
SBP	3 (42.9)	14 (19.2)	79 (29.9)	195 (50.1)	252 (52.2)	409 (67.3)	748 (89.8)
HYPER	2 (28.6)	4 (5.5)	48 (18.2)	89 (22.9)	139 (28.8)	176 (28.9)	429 (51.5)
TYPE2	2 (28.6)	17 (23.3)	86 (32.6)	129 (33.2)	217 (44.9)	344 (56.6)	677 (81.3)
SMK	0 (0.0)	17 (23.3)	122 (46.2)	243 (62.5)	365 (75.6)	496 (81.6)	809 (97.1)
FHCVD	3 (42.9)	28 (38.4)	164 (62.1)	304 (78.1)	405 (83.9)	560 (92.1)	830 (99.6)
BRA	1 (14.3)	10 (13.7)	28 (10.6)	44 (11.3)	63 (13.0)	86 (14.1)	150 (18.0)
BAF	2 (28.6)	22 (30.1)	64 (24.2)	99 (25.4)	141 (29.2)	216 (35.5)	427 (51.3)
RENAL	5 (71.4)	39 (53.4)	107 (40.5)	127 (32.6)	124 (25.7)	156 (25.7)	174 (20.9)

[a]Number of models that converged out of 1,000 samples.

Table A.1.5: Frequency of variable selection at varying EPV

EPV	Method	BMI	RATIO	SBP	AGE	SEX	All
2	BE(pval(0.05))	10.2	12.2	16.9	99.8	25.1	0.1
	BE(aic)	26.2	25.7	34.6	100.0	45.0	1.1
5	BE(pval(0.05))	19.0	14.6	30.2	100.0	53.0	0.0
	BE(aic)	33.6	28.7	47.2	100.0	71.8	2.0
10	BE(pval(0.05))	34.7	18.3	48.6	100.0	82.4	0.5
	BE(aic)	50.6	33.8	65.9	100.0	91.2	5.8
15	BE(pval(0.05))	42.0	22.1	60.7	100.0	93.7	1.6
	BE(aic)	57.9	37.7	78.9	100.0	98.0	10.9
20	BE(pval(0.05))	49.0	29.9	74.4	100.0	97.9	3.5
	BE(aic)	64.8	46.4	87.0	100.0	99.4	19.2
25	BE(pval(0.05))	60.1	28.3	79.2	100.0	99.7	7.2
	BE(aic)	76.0	45.0	90.1	100.0	99.9	25.6
50	BE(pval(0.05))	79.8	46.7	98.1	100.0	99.9	29.8
	BE(aic)	90.8	65.3	99.4	100.0	100.0	56.5

BE, backward elimination;
All, percentage of cases where all five main predictors are selected;
pval, p-value.

B.1. **Figures B.1.1 - B.1.3: Predictive accuracy measures**

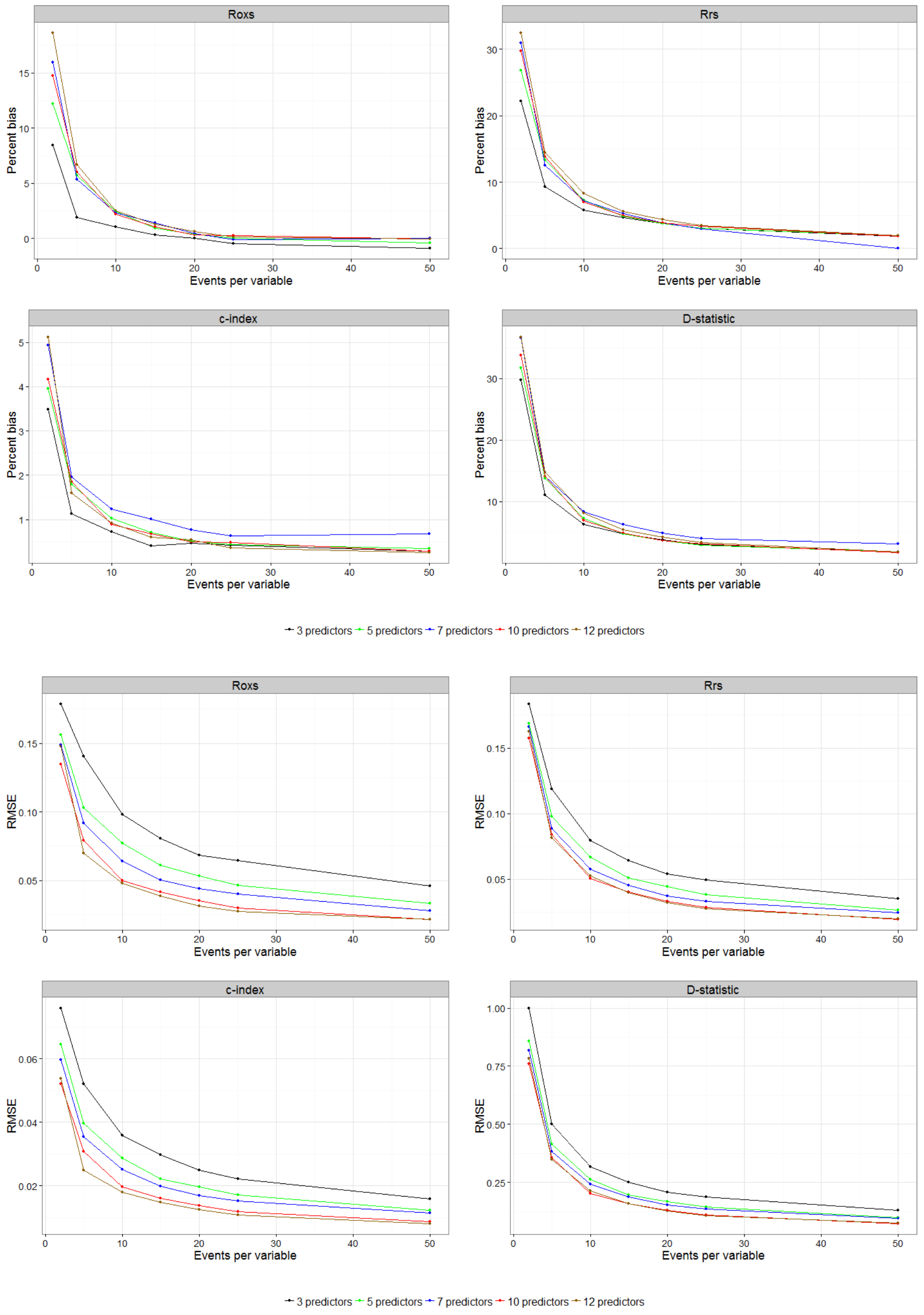

Figure B.1.1: Predictive accuracy measures for Cox models with varying EPV and number of covariates

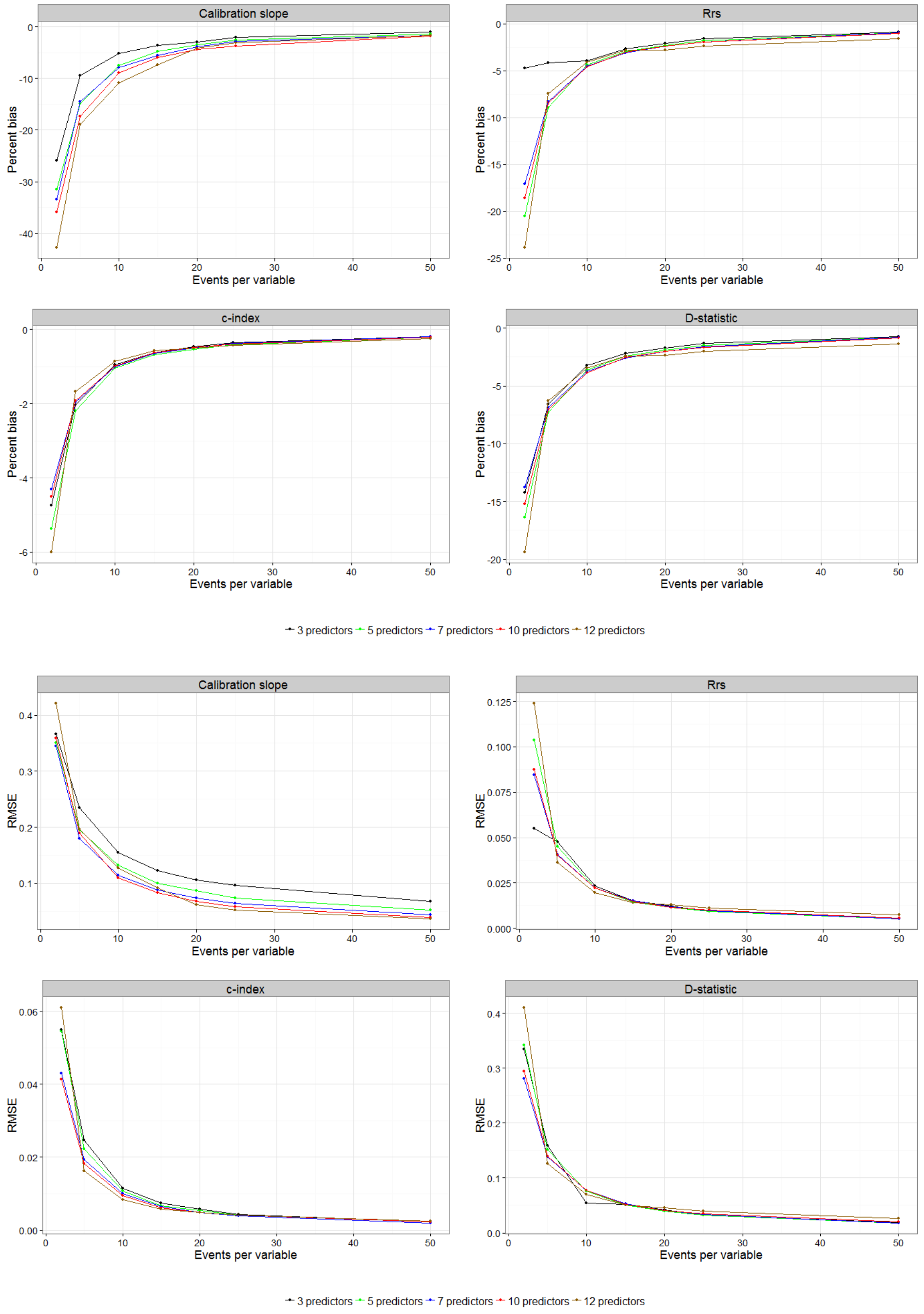

Figure B.1.2: Predictive accuracy measures for Cox models with varying EPV and number of covariates in external validation

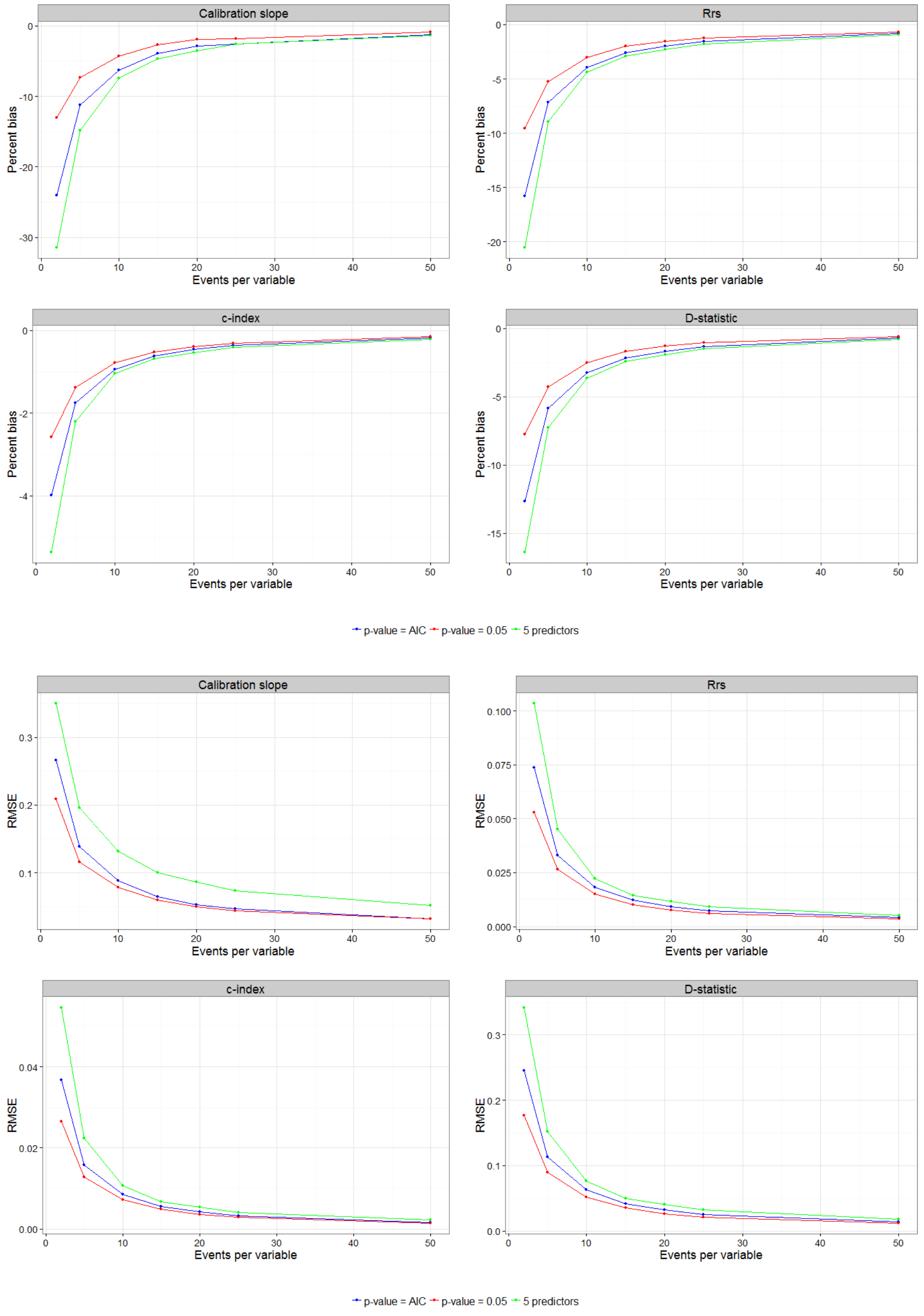

Figure B.1.3: Predictive accuracy measures for Cox models with varying EPV and number of covariates using variable selection

```
This is pdfTeX, Version 3.14159265-2.6-1.40.15 (TeX Live 2014/W32TeX)
(preloaded format=latex 2015.9.2)  31 JAN 2016 23:03
entering extended mode
 restricted \write18 enabled.
 %&-line parsing enabled.
**appendix.tex
(./appendix.tex
LaTeX2e <2014/05/01>
Babel <3.9k> and hyphenation patterns for 78 languages loaded.
(c:/texlive/2014/texmf-dist/tex/latex/base/article.cls
Document Class: article 2007/10/19 v1.4h Standard LaTeX document class
(c:/texlive/2014/texmf-dist/tex/latex/base/size12.clo
File: size12.clo 2007/10/19 v1.4h Standard LaTeX file (size option)
)
\c@part=\count79
\c@section=\count80
\c@subsection=\count81
\c@subsubsection=\count82
\c@paragraph=\count83
\c@subparagraph=\count84
\c@figure=\count85
\c@table=\count86
\abovecaptionskip=\skip41
\belowcaptionskip=\skip42
\bibindent=\dimen102
) (c:/texlive/2014/texmf-dist/tex/latex/tools/enumerate.sty
Package: enumerate 1999/03/05 v3.00 enumerate extensions (DPC)
\@enLab=\toks14
) (c:/texlive/2014/texmf-dist/tex/latex/amscls/amsthm.sty
Package: amsthm 2004/08/06 v2.20
\thm@style=\toks15
\thm@bodyfont=\toks16
\thm@headfont=\toks17
\thm@notefont=\toks18
\thm@headpunct=\toks19
\thm@preskip=\skip43
\thm@postskip=\skip44
\thm@headsep=\skip45
\dth@everypar=\toks20
) (c:/texlive/2014/texmf-dist/tex/latex/amsmath/amsmath.sty
Package: amsmath 2013/01/14 v2.14 AMS math features
\@mathmargin=\skip46
For additional information on amsmath, use the `?' option.
(c:/texlive/2014/texmf-dist/tex/latex/amsmath/amstext.sty
Package: amstext 2000/06/29 v2.01
(c:/texlive/2014/texmf-dist/tex/latex/amsmath/amsgen.sty
File: amsgen.sty 1999/11/30 v2.0
\@emptytoks=\toks21
\ex@=\dimen103
)) (c:/texlive/2014/texmf-dist/tex/latex/amsmath/amsbsy.sty
Package: amsbsy 1999/11/29 v1.2d
\pmbraise@=\dimen104
) (c:/texlive/2014/texmf-dist/tex/latex/amsmath/amsopn.sty
Package: amsopn 1999/12/14 v2.01 operator names
)
\inf@bad=\count87
LaTeX Info: Redefining \frac on input line 210.
\uproot@=\count88
```

```
\leftroot@=\count89
LaTeX Info: Redefining \overline on input line 306.
\classnum@=\count90
\DOTSCASE@=\count91
LaTeX Info: Redefining \ldots on input line 378.
LaTeX Info: Redefining \dots on input line 381.
LaTeX Info: Redefining \cdots on input line 466.
\Mathstrutbox@=\box26
\strutbox@=\box27
\big@size=\dimen105
LaTeX Font Info:    Redeclaring font encoding OML on input line 566.
LaTeX Font Info:    Redeclaring font encoding OMS on input line 567.
\macc@depth=\count92
\c@MaxMatrixCols=\count93
\dotsspace@=\muskip10
\c@parentequation=\count94
\dspbrk@lvl=\count95
\tag@help=\toks22
\row@=\count96
\column@=\count97
\maxfields@=\count98
\andhelp@=\toks23
\eqnshift@=\dimen106
\alignsep@=\dimen107
\tagshift@=\dimen108
\tagwidth@=\dimen109
\totwidth@=\dimen110
\lineht@=\dimen111
\@envbody=\toks24
\multlinegap=\skip47
\multlinetaggap=\skip48
\mathdisplay@stack=\toks25
LaTeX Info: Redefining \[ on input line 2665.
LaTeX Info: Redefining \] on input line 2666.
) (c:/texlive/2014/texmf-dist/tex/latex/amsfonts/amssymb.sty
Package: amssymb 2013/01/14 v3.01 AMS font symbols
(c:/texlive/2014/texmf-dist/tex/latex/amsfonts/amsfonts.sty
Package: amsfonts 2013/01/14 v3.01 Basic AMSFonts support
\symAMSa=\mathgroup4
\symAMSb=\mathgroup5
LaTeX Font Info:    Overwriting math alphabet `\mathfrak' in version
`bold'
(Font)                  U/euf/m/n --> U/euf/b/n on input line 106.
)) (c:/texlive/2014/texmf-dist/tex/latex/amsmath/amsxtra.sty
Package: amsxtra 1999/11/15 v1.2c
LaTeX Info: Redefining \nobreakspace on input line 65.
) (c:/texlive/2014/texmf-dist/tex/latex/comment/comment.sty
\CommentStream=\write3
Excluding comment 'comment') (c:/texlive/2014/texmf-
dist/tex/latex/graphics/gra
phicx.sty
Package: graphicx 2014/04/25 v1.0g Enhanced LaTeX Graphics (DPC,SPQR)
(c:/texlive/2014/texmf-dist/tex/latex/graphics/keyval.sty
Package: keyval 2014/05/08 v1.15 key=value parser (DPC)
\KV@toks@=\toks26
) (c:/texlive/2014/texmf-dist/tex/latex/graphics/graphics.sty
Package: graphics 2009/02/05 v1.0o Standard LaTeX Graphics (DPC,SPQR)
(c:/texlive/2014/texmf-dist/tex/latex/graphics/trig.sty
```

```
Package: trig 1999/03/16 v1.09 sin cos tan (DPC)
) (c:/texlive/2014/texmf-dist/tex/latex/latexconfig/graphics.cfg
File: graphics.cfg 2010/04/23 v1.9 graphics configuration of TeX Live
)
Package graphics Info: Driver file: dvips.def on input line 91.
(c:/texlive/2014/texmf-dist/tex/latex/graphics/dvips.def
File: dvips.def 2014/04/23 v3.0j Driver-dependant file (DPC,SPQR)
))
\Gin@req@height=\dimen112
\Gin@req@width=\dimen113
) (c:/texlive/2014/texmf-dist/tex/latex/psfrag/psfrag.sty
Package: psfrag 1998/04/11 v3.04 PSfrag (MCG)
\pfg@pcount=\count99
\pfg@scount=\count100
\pfg@temp=\write4
) (c:/texlive/2014/texmf-dist/tex/latex/tools/xspace.sty
Package: xspace 2009/10/20 v1.13 Space after command names (DPC,MH)
) (c:/texlive/2014/texmf-dist/tex/latex/tools/bm.sty
Package: bm 2004/02/26 v1.1c Bold Symbol Support (DPC/FMi)
\symboldoperators=\mathgroup6
\symboldletters=\mathgroup7
\symboldsymbols=\mathgroup8
LaTeX Font Info:    Redeclaring math alphabet \mathbf on input line 137.
LaTeX Info: Redefining \bm on input line 203.
) (c:/texlive/2014/texmf-dist/tex/latex/jknapltx/mathrsfs.sty
Package: mathrsfs 1996/01/01 Math RSFS package v1.0 (jk)
\symrsfs=\mathgroup9
) (c:/texlive/2014/texmf-dist/tex/latex/aries/setspace.sty
Package: setspace 2000/12/01 6.7 Contributed and Supported LaTeX2e
package
Package: `setspace' 6.7 <2000/12/01>
) (c:/texlive/2014/texmf-dist/tex/latex/multirow/multirow.sty
\bigstrutjot=\dimen114
) (c:/texlive/2014/texmf-dist/tex/latex/graphics/lscape.sty
Package: lscape 2000/10/22 v3.01 Landscape Pages (DPC)
) (c:/texlive/2014/texmf-dist/tex/latex/natbib/natbib.sty
Package: natbib 2010/09/13 8.31b (PWD, AO)
\bibhang=\skip49
\bibsep=\skip50
LaTeX Info: Redefining \cite on input line 694.
\c@NAT@ctr=\count101
) (c:/texlive/2014/texmf-dist/tex/latex/graphics/color.sty
Package: color 2014/04/23 v1.1a Standard LaTeX Color (DPC)
(c:/texlive/2014/texmf-dist/tex/latex/latexconfig/color.cfg
File: color.cfg 2007/01/18 v1.5 color configuration of teTeX/TeXLive
)
Package color Info: Driver file: dvips.def on input line 137.
(c:/texlive/2014/texmf-dist/tex/latex/graphics/dvipsnam.def
File: dvipsnam.def 2014/04/23 v3.0j Driver-dependant file (DPC,SPQR)
)) (c:/texlive/2014/texmf-dist/tex/latex/caption/caption.sty
Package: caption 2013/05/02 v3.3-89 Customizing captions (AR)
(c:/texlive/2014/texmf-dist/tex/latex/caption/caption3.sty
Package: caption3 2013/05/02 v1.6-88 caption3 kernel (AR)
Package caption3 Info: TeX engine: e-TeX on input line 57.
\captionmargin=\dimen115
\captionmargin@=\dimen116
\captionwidth=\dimen117
\caption@tempdima=\dimen118
```

```
\caption@indent=\dimen119
\caption@parindent=\dimen120
\caption@hangindent=\dimen121
)
\c@ContinuedFloat=\count102
) (c:/texlive/2014/texmf-dist/tex/latex/preprint/authblk.sty
Package: authblk 2001/02/27 1.3 (PWD)
\affilsep=\skip51
\@affilsep=\skip52
\c@Maxaffil=\count103
\c@authors=\count104
\c@affil=\count105
) (c:/texlive/2014/texmf-dist/tex/latex/float/float.sty
Package: float 2001/11/08 v1.3d Float enhancements (AL)
\c@float@type=\count106
\float@exts=\toks27
\float@box=\box28
\@float@everytoks=\toks28
\@floatcapt=\box29
) (c:/texlive/2014/texmf-dist/tex/latex/rotating/rotating.sty
Package: rotating 2009/03/28 v2.16a rotated objects in LaTeX
(c:/texlive/2014/texmf-dist/tex/latex/base/ifthen.sty
Package: ifthen 2001/05/26 v1.1c Standard LaTeX ifthen package (DPC)
)
\c@r@tfl@t=\count107
\rotFPtop=\skip53
\rotFPbot=\skip54
\rot@float@box=\box30
\rot@mess@toks=\toks29
) (c:/texlive/2014/texmf-dist/tex/latex/wrapfig/wrapfig.sty
\wrapoverhang=\dimen122
\WF@size=\dimen123
\c@WF@wrappedlines=\count108
\WF@box=\box31
\WF@everypar=\toks30
Package: wrapfig 2003/01/31  v 3.6
) (c:/texlive/2014/texmf-dist/tex/latex/aries/subfig.sty
Package: subfig 2005/06/28 ver: 1.3 subfig package
\c@KVtest=\count109
\sf@farskip=\skip55
\sf@captopadj=\dimen124
\sf@capskip=\skip56
\sf@nearskip=\skip57
\c@subfigure=\count110
\c@subfigure@save=\count111
\c@lofdepth=\count112
\c@subtable=\count113
\c@subtable@save=\count114
\c@lotdepth=\count115
\sf@top=\skip58
\sf@bottom=\skip59
) (./appendix.aux)
\openout1 = `appendix.aux'.

LaTeX Font Info:    Checking defaults for OML/cmm/m/it on input line 21.
LaTeX Font Info:    ... okay on input line 21.
LaTeX Font Info:    Checking defaults for T1/cmr/m/n on input line 21.
LaTeX Font Info:    ... okay on input line 21.
```

```
LaTeX Font Info:    Checking defaults for OT1/cmr/m/n on input line 21.
LaTeX Font Info:    ... okay on input line 21.
LaTeX Font Info:    Checking defaults for OMS/cmsy/m/n on input line 21.
LaTeX Font Info:    ... okay on input line 21.
LaTeX Font Info:    Checking defaults for OMX/cmex/m/n on input line 21.
LaTeX Font Info:    ... okay on input line 21.
LaTeX Font Info:    Checking defaults for U/cmr/m/n on input line 21.
LaTeX Font Info:    ... okay on input line 21.
Package caption Info: Begin \AtBeginDocument code.
Package caption Info: subfig package v1.3 is loaded.
Package caption Info: float package is loaded.
Package caption Info: rotating package is loaded.
Package caption Info: wrapfig package is loaded.
Package caption Info: End \AtBeginDocument code.
LaTeX Font Info:    Try loading font information for U+msa on input line
27.
(c:/texlive/2014/texmf-dist/tex/latex/amsfonts/umsa.fd
File: umsa.fd 2013/01/14 v3.01 AMS symbols A
)
LaTeX Font Info:    Try loading font information for U+msb on input line
27.
(c:/texlive/2014/texmf-dist/tex/latex/amsfonts/umsb.fd
File: umsb.fd 2013/01/14 v3.01 AMS symbols B
)
LaTeX Font Info:    Try loading font information for U+rsfs on input line
27.
(c:/texlive/2014/texmf-dist/tex/latex/jknapltx/ursfs.fd
File: ursfs.fd 1998/03/24 rsfs font definition file (jk)
)
Overfull \hbox (35.92314pt too wide) in paragraph at lines 52--54
 [][]
 []


Overfull \hbox (24.95654pt too wide) in paragraph at lines 76--78
 [][]
 []


LaTeX Warning: `!h' float specifier changed to `!ht'.


Overfull \hbox (20.25658pt too wide) in paragraph at lines 104--106
 [][]
 []


LaTeX Warning: `!h' float specifier changed to `!ht'.


Overfull \hbox (6.15668pt too wide) in paragraph at lines 133--135
 [][]
 []


LaTeX Warning: `!h' float specifier changed to `!ht'.
```

```
LaTeX Warning: `!h' float specifier changed to `!ht'.

[1

] [2] [3]

! LaTeX Error: Cannot determine size of graphic in biasengland.png (no
Bounding
Box).

See the LaTeX manual or LaTeX Companion for explanation.
Type  H <return>  for immediate help.
 ...

l.180 ...udegraphics[width = 5in]{biasengland.png}
                                                  }\\
Try typing  <return>  to proceed.
If that doesn't work, type  X <return>  to quit.

File: biasengland.png Graphic file (type eps)
<biasengland.png>

! LaTeX Error: Cannot determine size of graphic in rmseengland.png (no
Bounding
Box).

See the LaTeX manual or LaTeX Companion for explanation.
Type  H <return>  for immediate help.
 ...

l.181 ...udegraphics[width = 5in]{rmseengland.png}
                                                  }
Try typing  <return>  to proceed.
If that doesn't work, type  X <return>  to quit.

File: rmseengland.png Graphic file (type eps)
<rmseengland.png>

LaTeX Warning: Float too large for page by 81.73932pt on input line 184.


LaTeX Warning: `!h' float specifier changed to `!ht'.


! LaTeX Error: Cannot determine size of graphic in biasscotland.png (no
Boundin
gBox).

See the LaTeX manual or LaTeX Companion for explanation.
Type  H <return>  for immediate help.
 ...

l.189 ...degraphics[width = 5in]{biasscotland.png}
                                                  }\\
Try typing  <return>  to proceed.
If that doesn't work, type  X <return>  to quit.

File: biasscotland.png Graphic file (type eps)
```

```
<biasscotland.png>

! LaTeX Error: Cannot determine size of graphic in rmsescotland.png (no
Boundin
gBox).

See the LaTeX manual or LaTeX Companion for explanation.
Type  H <return>  for immediate help.
 ...

l.190 ...degraphics[width = 5in]{rmsescotland.png}
                                                  }
Try typing  <return>  to proceed.
If that doesn't work, type  X <return>  to quit.

File: rmsescotland.png Graphic file (type eps)
<rmsescotland.png>

LaTeX Warning: Float too large for page by 81.73932pt on input line 193.


LaTeX Warning: `h' float specifier changed to `ht'.


! LaTeX Error: Cannot determine size of graphic in biaspval.png (no
BoundingBox
).

See the LaTeX manual or LaTeX Companion for explanation.
Type  H <return>  for immediate help.
 ...

l.198 {\includegraphics[width = 5in]{biaspval.png}
                                                  }\\
Try typing  <return>  to proceed.
If that doesn't work, type  X <return>  to quit.

File: biaspval.png Graphic file (type eps)
<biaspval.png>

! LaTeX Error: Cannot determine size of graphic in rmsepval.png (no
BoundingBox
).

See the LaTeX manual or LaTeX Companion for explanation.
Type  H <return>  for immediate help.
 ...

l.199 {\includegraphics[width = 5in]{rmsepval.png}
                                                  }
Try typing  <return>  to proceed.
If that doesn't work, type  X <return>  to quit.

File: rmsepval.png Graphic file (type eps)
<rmsepval.png>

LaTeX Warning: Float too large for page by 81.73932pt on input line 202.
```

```
LaTeX Warning: `h' float specifier changed to `ht'.

[4

] [5] [6] [7] (./appendix.aux) )
Here is how much of TeX's memory you used:
 3856 strings out of 493118
 54253 string characters out of 6139011
 141204 words of memory out of 5000000
 7231 multiletter control sequences out of 15000+600000
 20735 words of font info for 79 fonts, out of 8000000 for 9000
 1141 hyphenation exceptions out of 8191
 36i,19n,38p,956b,465s stack positions out of
5000i,500n,10000p,200000b,80000s

Output written on appendix.dvi (7 pages, 14808 bytes).
```

LaTeX Source Files

Click here to download LaTeX Source Files: JCEV_revison2.tex
